# Bovine Adipocyte-Derived Exosomes Transport LncRNAs to Regulate Adipogenic Transdifferentiation of Bovine Muscle Satellite Cells

**DOI:** 10.3390/ani15233459

**Published:** 2025-11-30

**Authors:** Guangyao Meng, Jiasu Zhang, Zewen Wu, Jixuan Song, Qian Sun, Xinxin Zhang, Mengxia Sun, Yang Yi, Guangjun Xia

**Affiliations:** 1 Agriculture College, Yanbian University, Yanji 133002, China; mgy19971104@163.com (G.M.);; 2 Engineering Research Center of North-East Cold Region Beef Cattle Science & Technology Innovation, Ministry of Education, Yanbian University, Yanji 133002, China

**Keywords:** adipocytes, exosome lncRNA, muscle satellite cells, adipogenic transdifferentiation

## Abstract

Intramuscular fat content is a key factor in determining the quality and value of beef. Intramuscular adipocytes and satellite cells can interact with each other, and both are the source cells for intramuscular fat formation. To better understand the mechanism of bovine adipocytes regulating adipogenic transdifferentiation of muscle satellite cells (MSCs), this study established a co-culture system of bovine adipocytes and bovine MSCs, identified and isolated exosomes secreted by bovine adipocytes, co-cultured exosomes with bovine MSCs, and performed lncRNA sequencing of exosomes. The results showed that lipid droplets appeared in bovine MSCs under the co-culture system, and the expression levels of the PPARγ and CEBPA, which were lipogenesis-related genes, were significantly elevated. In addition, this study identified 3425 lncRNAs in adipocyte-derived exosomes, and the target genes of these lncRNAs were significantly enriched in gene functions and pathways related to transcriptional regulation, cellular differentiation, lipogenesis, and metabolism. It is worth noting that lncRNA-DGAT2 (lncDGAT2) can competitively bind to bta-miR-2455, increase the expression of target gene DGAT2, and promote adipogenic transdifferentiation of MSCs. In summary, bovine adipocytes can regulate the adipogenic transdifferentiation of MSCs through exosomes and exosomal lncRNAs. This study provides new insights into the regulation mechanism of bovine intramuscular fat deposition.

## Simple Summary

Intramuscular fat is the material basis for beef marbling. Unfortunately, intramuscular fat formation is complex, and isolated cell culture experiments cannot adequately simulate in vivo conditions to properly analyze this process. Given that exosomal bioactive factors are known regulators of lipid metabolism, this study aimed to investigate whether adipocyte-derived lncRNAs from Yanbian yellow cattle could modulate the adipogenic differentiation of muscle satellite cells in a co-culture system. This study unveiled a novel regulatory mechanism in adipogenic transdifferentiation mediated by exosomal lncRNA-DGAT2 derived from bovine adipocytes. Specifically, exosomal lncRNA-DGAT2 competitively binds to the muscle-specific microRNA bta-miR-2455, thereby upregulating the expression of the adipogenic gene DGAT2 and inducing adipogenic differentiation in muscle satellite cells (MSCs). These findings elucidate the complex crosstalk between adipose and muscle tissues and provide valuable insights into the molecular mechanisms governing intramuscular fat deposition in beef cattle. Understanding the role of exosomal lncRNAs in adipogenic transdifferentiation not only advances our knowledge of bovine genetics but also holds potential implications for improving meat quality traits and breeding strategies in the beef cattle industry.

## 1. Introduction

In production animals, adipose tissue plays a crucial role in the determination of meat quality [1,2]. In the mammalian body, the main adipose depots are subcutaneous, visceral, intramuscular, and intermuscular fat [3]. Intramuscular fat is primarily distributed between muscle bundles and muscle fibers [4]. Its varying quantity and distribution contribute to the marbling patterns seen in meat. IMF can sever the cross-linking structures between muscle fiber bundles, facilitating the breaking of muscle fibers during the chewing of meat, thereby enhancing its tenderness [5]. The juiciness of meat depends on its moisture content and the saliva secreted during chewing [6]. IMF stimulates saliva secretion [7], thereby enhancing the meat’s juiciness. The fat-soluble components contained within intramuscular fat and their degradation products (such as aldehydes, alcohols, and ketones) can enhance the flavor of meat [6]. During cooking, the unsaturated fatty acids (UFAs) contained in IMF undergo oxidation to produce volatile compounds that enhance the flavor of the meat [8]. The intramuscular fat content is one of the most important determinants of meat quality. However, enhancing IMF deposition without increasing the other three adipose depots is one of the main challenges faced by the meat industry [1,3]. The deposition of intramuscular fat relied on processes such as preadipocyte differentiation and proliferation within muscle cells, all regulated by various molecular factors [9,10,11]. MSCs possess the potential to differentiate into adipocytes and can differentiate into adipocytes under the influence of various factors [12,13,14,15]. Notably, preadipocytes can increase the expression of adipogenic genes and lipid deposition in MSCs [16,17]. Therefore, investigating the role of preadipocytes on MSCs’ adipogenic differentiation and delving into the underlying mechanisms is an innovative and valuable avenue of research.

Exosomes are a class of membranous vesicles with diameters of 40–200 nm, which can be secreted by a variety of cells and exist stably in body fluids [18,19]. As novel mediators of intercellular and inter-tissue communication, exosomes extensively participate in regulating lipid metabolism within the animal organism through the bioactive factors present within them [20]. Certain circulating miRNAs within exosomes can suppress PPAR-γ expression by binding to the 3′ untranslated region (3′ UTR) of target genes, thereby influencing lipid synthesis [21,22]. Exosomes can influence lipolysis by carrying phospho-hormone sensitive lipase (P-HSL), adrenomedullin, and modulating the TGFβ signaling pathway [23,24,25]. Moreover, exosomes also possess a function in lipid transport, capable of directly transferring lipids from parent cells to recipient cells, such as cholesterol, fatty acids, eicosanoids, etc. [26,27,28]. In vitro experiments by researchers confirmed that skeletal muscle cells can absorb adipose-derived exosomes [29]. It is found that adipose tissue-derived exosome adiponectin can regulate the differentiation of C2C12 cells [30]. Therefore, adipocytes may regulate muscle cell differentiation via exosomes. Long non-coding RNAs (lncRNAs) are non-coding transcripts exceeding 200 nt in length, the majority of lncRNAs exhibit lower conservation across species compared to mRNA sequences encoding the proteome [31]. LncRNAs are involved in the mechanisms of adipose tissue deposition by influencing adipogenesis and lipid metabolism [32]. LncRNA can influence fat deposition by affecting adipocyte differentiation, for example, lncRNA-000414 inhibits the proliferation of intramuscular adipocytes in pigs [33]. Lnc-U90926 was found to be predominantly expressed in adipose tissue, and the expression of Lnc-U90926 inhibited 3T3-L1 preadipocyte differentiation to adipose tissue by decreasing the mRNA levels of PPARγ, adipose tissue acid-binding protein 4 (FABP4), and adiponectin, and the protein levels of PPARγ and FABP4 [34]. Moreover, lncRNAs can regulate lipid metabolism to influence fat deposition, for example, the lncRNA-SRA limits adipose triglyceride lipase promoter activity in hepatic steatosis primarily by inhibiting forkhead box protein O1 (FoxO1) expression [35]. The overexpression of lncHR1 blocked the expression of sterol regulatory element binding protein (SREBP-1c) and fatty acid synthase and suppressed the accumulation of triglycerides and lipid droplets in hepatocytes induced by oleic acid [36]. With the deepening understanding of lncRNAs, it has been found that lncRNAs can exist in the nucleus, cytoplasm, and extracellular vesicles [37]. Notably, lncRNAs and exosome lncRNAs exert lipid metabolism regulatory functions by competitively binding to miRNAs. For example, Guo et al. found that lncHLEF not only acts as a competitive endogenous RNA to regulate the miR-2188-3p/GATA6 axis, but also can be delivered to intramuscular and abdominal preadipocytes via exosomes secreted by chicken hepatocytes to enhance the differentiation of chicken intramuscular preadipocytes without altering the differentiation of chicken abdominal preadipocytes, which in turn increases intramuscular fat deposition in chickens [38]. These advances underscore the pivotal role of lncRNAs carried by exosomes in animal lipid biogenesis and metabolism. Unfortunately, the function of bovine preadipocyte-secreted exosomal lncRNAs and their impact on MSCs remain unclear.

In this study, we proposed to construct a co-culture system of bovine adipocytes and MSCs to simulate the in vivo environment of intramuscular fat formation. RNA-seq technology and bioinformatics analysis were used to screen adipocyte-derived exosome lncRNAs. Validation was conducted through a series of cellular and molecular biology experiments. In this study, exosomes and exosomal lncRNAs derived from bovine adipocytes were studied to explore the molecular functions of exosomes and exosomal lncRNAs in bovine muscle satellite cells and their regulatory mechanisms on adipogenic transdifferentiation of MSCs. Furthermore, this study will supplement the research on intercellular communication mechanisms and provide new insights and a theoretical basis for the molecular mechanism of intramuscular fat deposition.

## 2. Materials and Methods

### 2.1. Ethics Statement

Animal care and experimentation were conducted in accordance with the guidelines outlined in the “Regulations for the Administration of Affairs Concerning Experimental Animals” (2004) by the Ministry of Science and Technology of China. The study protocol was also approved by the Medical Ethics Committee of Yanbian Hospital, Affiliated to Yanbian University (Approval No: 201702).

#### Sample Collection

Three one-week-old healthy and disease-free Yanbian yellow cattle calves were selected. Subcutaneous adipose tissue and longissimus dorsi muscle tissue were collected under sterile conditions. The collected tissues were immersed in pre-cooled phosphate-buffered saline (PBS) and quickly placed in an ice box. After disinfection, it was brought back to the cell culture laboratory for cell separation.

### 2.2. Isolation, Culture, and Differentiation of Bovine Preadipocytes

Bovine primary preadipocytes were isolated by enzyme digestion. The collected subcutaneous adipose tissue was washed several times with PBS, and the fibers and blood vessels in the adipose tissue were removed. After being immersed in the 75% alcohol for 10 min, the adipose tissue was washed three times with PBS, and the adipose tissue was cut into a paste and incubated with type IV collagenase in a 37 °C-water bath for 60 min. Digestion was then terminated by adding an equal volume of growth medium to the digestion solution. After filtration (70, 100 μm nylon mesh) and centrifugation (1500× *g*, 10 min), cell precipitates were resuspended in a growth medium and cultured in a cell incubator (37 °C, 5% CO_2_). The bovine preadipocytes were transplanted into 6-well plates, and the cells were observed at regular intervals. When the cells reached 80% confluence, the medium was replaced with an equal volume of differentiation medium (induction medium I containing 10 μg/mL insulin, 0.5 mM IBMX, and 1.0 μM DEX). The morphological changes of the cells were observed under a microscope the next day, and then the cells were further cultured in an induction medium II. Two days later, the morphological changes of the cultured cells were observed under a low-power microscope, the induction medium II was discarded, and the cells were cultured with growth medium again until the 9th day of induction. The degree of differentiation of preadipocytes was evaluated by oil red 0 staining and triglyceride content determination. The expression of adipogenic-related marker genes in preadipocytes was detected by RT-qPCR.

### 2.3. Isolation, Culture, and Differentiation of Bovine MSCs

Bovine primary MSCs were isolated by the tissue culture method. The collected longissimus dorsi muscle tissue was washed several times with PBS, and the fibers and blood vessels in the muscle tissue were removed. The muscle tissue was immersed in 75% alcohol for 10 min and then rinsed with PBS three times. The longissimus dorsi muscle tissue was cut into pieces of about 1 mm^3^. After rinsing with PBS, the samples were inoculated into a culture flask; then, an appropriate amount of growth medium was added to the culture flask and cultured in a cell incubator (37 °C, 5% CO_2_). When the cells were confluent to about 70%, the tissue blocks were discarded. When the cells were confluent to 80%, the adherent cells were digested with trypsin. Then, the cells were purified by differential adhesion using a culture flask with 0.01% polylysine, and the purified cells were inoculated into a common culture flask. The RT-qPCR was used to detect the expression of myogenic marker genes in MSCs.

### 2.4. Oil Red O Staining and Determination of Triglyceride Content

Before induction of differentiation and on day 9 after induction of differentiation, adipocytes were subjected to Oil red O staining and determination of triglyceride content. Briefly, the cells were rinsed with PBS, and the cell morphology was fixed with formaldehyde solution at a concentration of 4%; the cells were then reacted with Oil Red O for 30 min, and the staining results were observed under a light microscope and measured photometrically using an enzyme marker (Spark, Tecan, Männedorf, Switzerland). Triglyceride content was determined according to the instructions of the TG assay kit (Blue Sky, Shanghai, China).

### 2.5. Immunofluorescence

The 4th passage of cattle MSCs was seeded at a density of 1 × 105 cells per well in a 6-well plate. When the cells reached approximately 80% confluence, the culture medium was removed, and the cells were washed three times with PBS. They were then fixed with 4% paraformaldehyde at room temperature for 30–40 min. Next, 0.1% Triton X-100 was added at room temperature for 1 h to facilitate permeabilization. Subsequently, BSA blocking solution was added, and the cells were incubated at 37 °C for 2 h. After blocking, the blocking solution was removed, and 1 mL of primary antibodies against Myogenic Differentiation Antigen1 (MyoD1) and Myostatin (MSTN), diluted at a ratio of 1:200, was added to each well. The cells were incubated at 37 °C for 2 h. After incubation, the primary antibodies were washed off, and a secondary antibody labelled with FITC at a dilution of 1:50 was added. The cells were incubated at 37 °C for 2 h. Following incubation, the cells were washed with PBS containing 2% BSA to remove excess secondary antibodies. DAPI staining was performed for 15 min to visualize the cell nuclei. The results were observed and photographed under a fluorescence microscope (BX53; Olympus, Tokyo, Japan).

### 2.6. Total RNA Extraction and Real-Time Fluorescence Quantitative PCR

This experiment employed fifth-generation bovine preadipocytes and fifth-generation bovine myoblasts as test materials. The relative expression levels of adipogenesis marker genes in preadipocytes and myogenesis marker genes in myoblasts were detected using RT-qPCR. In this experiment, specific primers for RT-qPCR were designed using Primer Premier 5.0 and Oligo 7.0 software, with the mRNA primer sequences listed in Table S1. The RNA was extracted from cells using the Eastep Super Total RNA Extraction Kit (Promega, Shanghai, China). And reverse transcribed into cDNA using the FastKing gDNA Dispelling RT SuperMix kit (TIANGEN, Beijing, China). The cDNA was prepared by qRT-PCR using the SuperReal PreMix Plus kit (TIANGEN, Beijing, China). The RT-PCR was performed using the SuperReal PreMix Plus kit (TIANGEN, Beijing, China). The RT-qPCR reaction solutions were prepared on ice; the reaction system is shown in Table 1. The qPCR reaction was performed on a PCRmax Eco 48 real-time PCR instrument (PCRmax, Stone, Staffordshire, UK) with the following procedure: pre-denaturation at 95 °C for 15 min, denaturation at 95 °C for 10 s, and annealing at 60 °C for 30 s, for a total of 40 cycles. All the experiments were carried out three times independently. Relative mRNA expression was calculated using the 2^−ΔΔCt^ method, and β-actin was used as a housekeeping gene to regulate the expression of other protein-coding genes.

### 2.7. Protein Extraction and Western Blot Analysis

Total protein was extracted by the RIPA Lysis Buffer (Beyotime, Beijing, China) with 1 mM phenylmethanesulfonyl fluoride (Beyotime, Beijing, China). The total protein concentration of the cell lysates was determined by the Enhanced BCA Protein Assay Kit (Beyotime, China) according to the manufacturer’s instructions. Protein samples were electrophoresed using 10% sodium dodecyl sulfate-polyacrylamide gel electrophoresis gels (Solarbio, Beijing, China) with 20 μg of protein per well, and a polyvinylidene fluoride (PVDF) membrane (Millipore, Billerica, MA, USA) of the same area as the gel was cut. The PVDF membrane was activated in methanol for 30 s and then put into the membrane transfer solution, and the membrane was transferred at constant pressure and 0.2 A. After completion, the PVDF membrane was rinsed with TBS containing 0.1% Tween 20 (TBST) and blocked with TBST containing 5% ovalbumin (Solarbio, Beijing, China) on a shaker at 22 ± 3 °C for 2 h. The PVDF membrane was then incubated overnight with the primary antibody at 4 °C in a blocking buffer and washed five times with TBST. And then incubated with the horseradish peroxidase-conjugated secondary antibody for 2 h at 4 °C. The PVDF membrane was washed with TBST, and the immunoblots were prepared using the chemiluminescent solutions and analyzed using the Alliance MINI HD9 AUTO Western Blot Imaging System (UVITEC, Cambridge, UK). The intensities of the target protein bands were normalized as the intensity of the actin bands and calculated via the ImageJ 1.54 program. All experiments were conducted in 3 replicates.

### 2.8. Co-Culture System Establishment

Adipocytes and MSCs were co-cultured to investigate their interactions and molecular processes under specific conditions. The fifth-generation bovine adipocytes and the fifth-generation bovine MSCs were co-seeded in a transwell culture system at a 1:2 ratio. The transwell system consisted of inserts with a polycarbonate membrane with a 0.4 μm pore size. The growth medium was replaced every 2 d. During medium exchange, sterile forceps were used to carefully remove the transwell inserts, and 2 mL of growth medium was added to each well of a 6-well plate. The transwell inserts nested within the wells of the 6-well plate were returned to the plate. Next, 1 mL of growth medium was added to each upper chamber of the transwell inserts. The entire setup was placed in an incubator for continuous cultivation. After 5 d of co-culture, the experiment was terminated for subsequent assays, including total RNA and protein extraction. The MSCs before and after co-culture were subjected to RT-qPCR, Western blot, Oil Red O staining, and triglyceride content determination.

### 2.9. Adipocyte-Derived Exosomes Extraction, Characterization and Co-Culture with MSCs

The fifth-generation bovine adipocytes were cultured in DMEM complete medium with 10%, 5%, and 3% serum to consume fetal bovine serum with exosomes. After 10 h of culture, the medium was discarded, and the adipocytes were washed three times with PBS. Then the adipocytes were cultured in DMEM complete medium containing 10% exosome-free fetal bovine serum, and 1 mL of the serum sample was centrifuged at 2000× *g* for 30 min. The supernatant was centrifuged at 10,000× *g* for 45 min to isolate the larger vesicles. The supernatant was taken, and the filtrate was collected through a 0.45 μm filter membrane. The filtrate was ultracentrifuged at 100,000× *g* for 70 min, and after resuspending the precipitate with 10 mL of pre-cooled 1 × PBS, the supernatant was again ultracentrifuged at 100,000× *g* for 70 min, then the supernatant was removed and resuspended with 100 μL of pre-cooled 1 × PBS to obtain the exosome suspension. All centrifugation procedures were performed at 4 °C.

Transmission electron microscopy (TEM) (N30E, NanoFCM, Xiamen, China) was used to visualize the exosome morphology, and nanoparticle tracking analysis (NTA) was used to determine the concentration and size distribution of exosomes. The expression levels of marker proteins in the isolated extracellular vesicles were determined. Then, 100 μL adipose-derived exosomes were added to the fifth-generation bovine MSCs culture medium for co-culture for 5 days, and RT-qPCR and Western blot tests were performed on MSCs.

### 2.10. Exosomal LncRNA-Seq

The extracted total RNA of adipocyte-derived extracellular vesicles was sent to Xiamen Life Link Biotechnology Company (Xiamen, China) for lncRNA sequencing. The lncRNA-seq in this study was conducted using the Illumina platform (ThermoFisher Science Inc., Waltham, MA, USA), with the reference genome being the complete genome sequence of cattle (ARS-UCD1.2) from the NCBI database. To ensure data quality, we performed quality control on the raw reads using Fastp (v0.23.1) (https://github.com/OpenGene/fastp) (accessed on 7 February 2022), filtering out low-quality data to obtain clean reads. Next, we employed the Hisat2 software (2.2.1) to conduct reference genome-based alignment analysis. Transcript construction was performed using Stringtie (2.2.0) software with alignment files. Comparison with genomic annotation files via Gffcompare (v0.11.4) yielded annotated and candidate lncRNA transcripts. Transcripts exceeding 200 nt in length were selected as candidate lncRNA transcripts. Four software packages—CPC2 (3.18), CNCI (3.0), Pfam (33.1), and FEElnc (v0.2.1) to predict coding potential. Transcripts predicted by at least three software packages were filtered to identify novel lncRNAs. Quantification and annotation of lncRNAs were performed using featureCounts (2.0.3). The prediction of target genes for lncRNAs was performed based on the cis-regulatory mode.

### 2.11. Cell Transfection

When the MSCs in the culture bottle reached 80% confluence, they were digested with 0.25% trypsin and counted. The cells were then seeded at a density of 1 × 106 cells/well in a 6-well cell culture plate. The plate was placed in a 37 °C, 5% CO_2_ humidified incubator for cultivation. Once the cells in the wells reached 80% confluence, the medium was replaced with 2 mL serum-containing medium. Meanwhile, the transcript sequence of lncDGAT2 (XR_237845.3) was synthesized using the NCBI database. After considering factors such as vector resistance, selection markers, and promoter sequences, NheI (5′-G/CTAGC-3′) and EcoRI (5′-G/AATTC-3′) restriction enzymes were chosen for connecting the lncDGAT2 sequence to the overexpression vector. Sequencing of the overexpression vector confirmed successful construction of the lncDGAT2 carrier, with the synthesized lncDGAT2 transcript sequence fully integrated into the vector without any peaks or anomalies. Transfection was then performed according to the instructions provided by the Lipofectamine 2000 ((Invitrogen, Carlsbad, CA, USA) transfection reagent kit. Briefly, Lipofectamine 2000 was added to two sterile 1.5 mL microcentrifuge tubes and mixed thoroughly with the appropriate amount of Opti-MEM. An appropriate amount of lncDGAT2 expression vector (overexpression vector [pcDNA3.1-lncDGAT2] or small interfering RNA [siRNA] [si-lncDGAT2]; empty vectors [pcDNA3.1-NC and si-NC] were used as controls) was added to another tube and mixed thoroughly with Opti-MEM. The liquids from the two tubes were then combined and mixed thoroughly using a pipette to combine the liposomes and the lncRNA expression vector. This mixture was incubated at room temperature for 5 min to form liposome complexes. The prepared complex was evenly added to the cells in the culture plate, and the plate was again incubated at 37 °C and 5% CO_2_. After 48 h, the cell status was observed, and subsequent experimental procedures were performed.

### 2.12. Dual-Luciferase Reporter Gene Assay

Utilizing the TargetScan algorithm, potential binding sites of Bta-miR-2455 and DGAT2 were identified. The target sequences of lncDGAT2 and DGAT2 3′UTR were subjected to BLAST (https://blast.ncbi.nlm.nih.gov/Blast.cgi) (accessed on 16 September 2022) alignment to locate the binding sequence between lncDGAT2 and Bta-miR-2455. Plasmids containing both wild-type and mutant constructs of lncRNA, incorporating this identified sequence, were constructed for the dual luciferase reporter assay. These plasmids were co-transfected with synthetic analogs of Bta-miR-2455 and a negative control into HepG2 cells. After 48 h, the cell culture medium was discarded, and 200 μL of cell lysis buffer was added to each well. Incubation at room temperature for 10 min facilitated comprehensive cell lysis. The resulting lysate was centrifuged at 10,000× *g* for 5 min, and the supernatant was collected as the test sample. The luciferase assay substrate for Renilla luciferase (100×) was thawed and set aside. The chemiluminescence instrument was initiated in accordance with the instructions. Briefly, 100 μL of the collected lysate supernatant was dispensed into each well of a 96-well luminescence plate. Subsequently, 100 μL of firefly luciferase assay working solution was added, followed by gentle aspiration to ensure thorough mixing. Luminescence readings were taken with a 5 s duration. Following this, 100 μL of Renilla luciferase assay working solution was introduced, mixed through aspiration, and luminescence readings were again taken over 5 s. Data processing involved using the luminescence value from the Renilla luciferase as the reference, calculating the ratio of luminescence values obtained from the firefly luciferase assay relative to the Renilla luciferase assay, thereby assessing the degree of activation of the target reporter gene.

### 2.13. Data Statistics and Analysis

All results are presented as “mean ± standard deviation.” Statistical analysis and data analysis for experiments such as RT-qPCR, Western blot (WB), and triglyceride measurements were performed using SPSS 20 software. Independent sample *t*-tests were employed to analyze differences between the two groups, with “*p* < 0.05” indicating statistically significant differences. GraphPad Prism 8.0 software was utilized for generating statistical graphs related to experiments such as RT-qPCR, WB, and triglyceride measurements.

## 3. Results

### 3.1. Morphology, Differentiation, and Identification of Bovine Precursor Adipocytes

**Figure 1:**
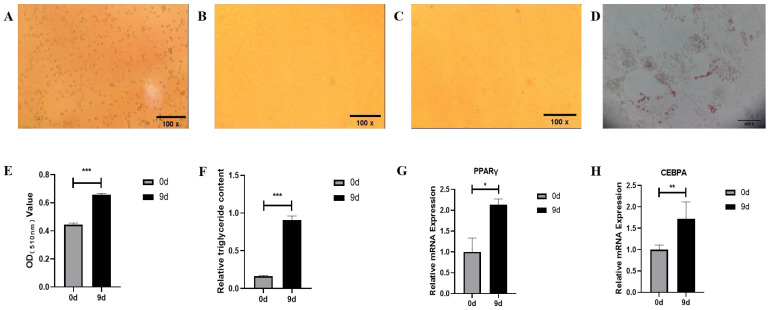
Identification of bovine primary adipocytes: (**A**) Morphology of freshly seeded preadipocytes (100×). (**B**) Morphology of preadipocytes seeded for 2 days (100×). (**C**) Morphology of preadipocytes seeded for 6 days (100×). (**D**) Oil red O staining of preadipocytes (400×) (Red indicates lipid droplets). (**E**) The OD value of oil red O staining before and after preadipocyte differentiation. (**F**) Triglyceride content before and after preadipocyte differentiation. (**G**) Expression of the PPARγ gene before and after preadipocyte differentiation. (**H**) Expression of the CEBPA gene before and after preadipocyte differentiation; mean ± SEM, *n* = 3, “*”, “**” and “***” represent *p* < 0.05, *p* < 0.01 and *p* < 0.001, respectively.

The isolated preadipocytes were inoculated into the cell culture bottle (Figure 1A), and the adherent cells began to grow gradually after 2 days of culture. During the culture process, the cells were arranged according to certain rules and grew in the same direction (Figure 1B,C). The Oil red O staining showed that after induction of differentiation up to day 9, small red, rounded lipid droplets were distributed around the adipocytes, and the differentiated adipocytes had an increased content of lipid droplets compared to the precursor adipocytes (Figure 1D,E). Triglyceride content measurements showed that the triglyceride content of adipocytes after 9 days of differentiation was significantly higher than that of the undifferentiated precursor adipocytes (Figure 1F). Meanwhile, RT-qPCR results showed that with the differentiation of adipocytes, the relative expression of the Peroxisome proliferator-activated receptors γ (PPARγ) gene was significantly upregulated (*p* < 0.05) and the relative expression of the CCAAT/enhancer binding protein alpha gene (CEBPA) gene was highly significant upregulated (*p* < 0.01), as compared with that of the precursor adipocytes before differentiation (Figure 1G,H). These results indicate that the samples of bovine preadipocytes and adipocytes can meet the requirements of subsequent experiments.

### 3.2. Morphology, Differentiation, and Identification of Bovine MSCs

**Figure 2:**
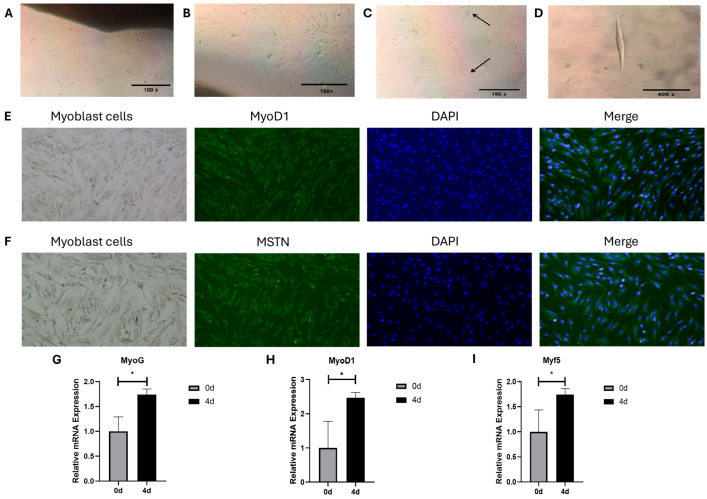
Identification of bovine primary MSCs: (**A**) Cell morphology on day 4 of tissue block culture (100×). (**B**) Cell morphology on day 8 of tissue block culture (100×). (**C**) Cell morphology on day 12 of tissue block culture (100×), (The arrows indicate observable myoblasts.). (**D**) Cell morphology of a single MSC (400×). (**E**) MyoD1 immunofluorescence staining of MSCs, proliferating cells are labelled green, while all cell nuclei are labelled blue. (**F**) MSTN immunofluorescence staining of MSCs, proliferating cells are labelled green, while all cell nuclei are labelled blue. (**G**) Expression of the MyoG gene before and after MSC differentiation. (**H**) Expression of the MyoD1 gene before and after MSC differentiation. (**I**) Expression of the Myf5 gene before and after MSC differentiation; mean ± SEM, *n* = 3, “*” represent *p* < 0.05.

The results of microscopic observation showed that the bovine MSCs free from the tissue blocks were spindle or spindle-shaped (Figure 2A,B). During the culture process, most of the MSCs began to converge in the same direction and gradually converged into myotubes (Figure 2C,D). Immunofluorescence results showed that the myogenesis-related genes MyoD1 and MSTN expressed immunopositive products in MSCs (Figure 2E,F). Meanwhile, the results of RT-qPCR showed that the expression levels of myogenic marker genes Myogenic factor 5 (Myf5), MyoD1, and Myogenin (MyoG) were significantly elevated in MSCs at the time of differentiation up to day 4 (4 d) compared to the pre-differentiation period (0 d) (*p* < 0.05) (Figure 2G–I). These results indicate that the isolated bovine MSCs have the potential for differentiation and can meet the requirements of subsequent experiments.

### 3.3. Lipogenic Transdifferentiation of MSCs in Co-Culture System

**Figure 3:**
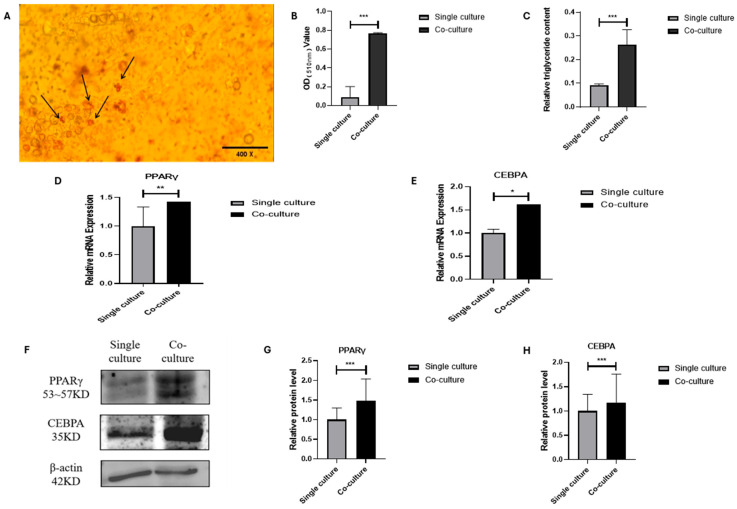
Adipogenic transdifferentiation of MSCs under the co-culture system: (**A**) Oil red O staining of MSCs under the co-culture system (400×). The red portion indicated by the arrow represents lipid droplets. (**B**) The OD value of oil red O staining of MSCs before and after co-culture. (**C**) The content of triglyceride (TG) in MSCs before and after co-culture. (**D**,**E**) The mRNA expression of PPARγ and CEBPA in MSCs before and after co-culture. (**F**–**H**) The protein expression of PPARγ and CEBPA in MSCs before and after co-culture; mean ± SEM, *n* = 3, “*”, “**” and “***” represent *p* < 0.05, *p* < 0.01 and *p* < 0.001, respectively.

Bovine adipocytes and MSCs were co-cultured, and the results of oil red O staining showed that fat droplets appeared in MSCs (Figure 3A,B). And the results of triglyceride content measurement showed that the triglyceride content in cocultured MSCs was significantly increased compared with that in the single culture (Figure 3C). In addition, the expression of lipogenesis-related genes and proteins in the MSCs after co-culture was identified. The results showed that the expression of lipogenesis-related gene PPARγ was highly significantly increased (*p* < 0.01) and CEBPA was significantly increased (*p* < 0.05) in MSCs (Figure 3D,E), and the protein expression of both genes was highly significantly increased (*p* < 0.001) (Figure 3F–H). These results showed that the lipid production in bovine MSCs increased after co-culture with bovine adipocytes, which showed a phenomenon of lipogenic transdifferentiation.

### 3.4. Identification and Role of Bovine Adipocyte-Derived Exosomes

**Figure 4:**
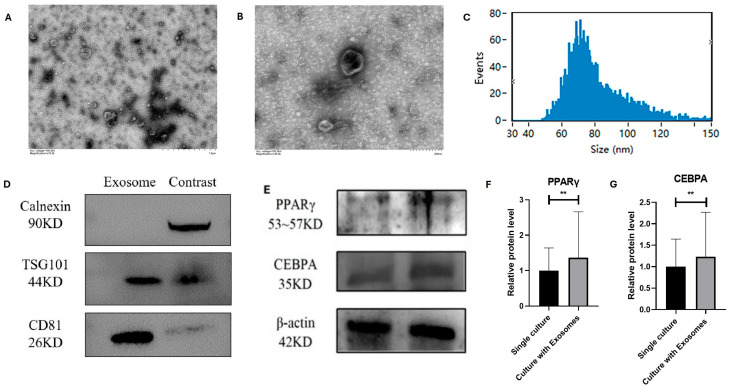
Adipogenic transdifferentiation of MSCs under co-culture conditions: (**A**,**B**) Electron microscopy images of extracellular vesicles from adipocytes. (**C**) Particle size distribution of adipocyte-derived extracellular vesicles. (**D**) Protein expression of important exosome markers. (**E**–**G**) Protein expression of PPARγ and CEBPA after co-culturing with adipocyte-derived extracellular vesicles; mean ± SEM, *n* = 3, “**” represents *p* < 0.01.

The morphology of extracellular vesicles derived from bovine adipocytes was observed by transmission electron microscopy, and the results showed that the vesicles had distinct membrane boundaries and were cup-shaped in different sizes (Figure 4A,B). The particle size of the isolated extracellular vesicles ranged from 40 to 150 nm, with an average particle size of 80.34 nm (Figure 4C). The results showed that the extracellular vesicles isolated in this experiment were morphologically consistent with the morphological definition of exosomes, and could be used in the next step of the exosome-related quality control work. Then, the protein expression of the markers of isolated extracellular vesicles was detected, and the results showed that compared with the control group (adipocytes), the positive markers TSG101 and CD81 of extracellular vesicles had higher protein expressions, while the negative marker Calnexin was almost not expressed (Figure 4D). Therefore, the expression of extracellular vesicle marker protein was consistent with that of exosomal marker protein.

Adipocyte-derived exosomes were added to the culture medium of MSCs for 5 days, and the protein immunoblotting test of MSCs showed that the protein expressions of PPARγ and CEBPA genes in MSCs were significantly upregulated (*p* < 0.01) (Figure 4E,F), indicating that adipocyte-derived exosomes promoted the expression of lipogenic genes in MSCs.

### 3.5. LncRNA-Seq of Adipocyte-Derived Exosomes

**Figure 5:**
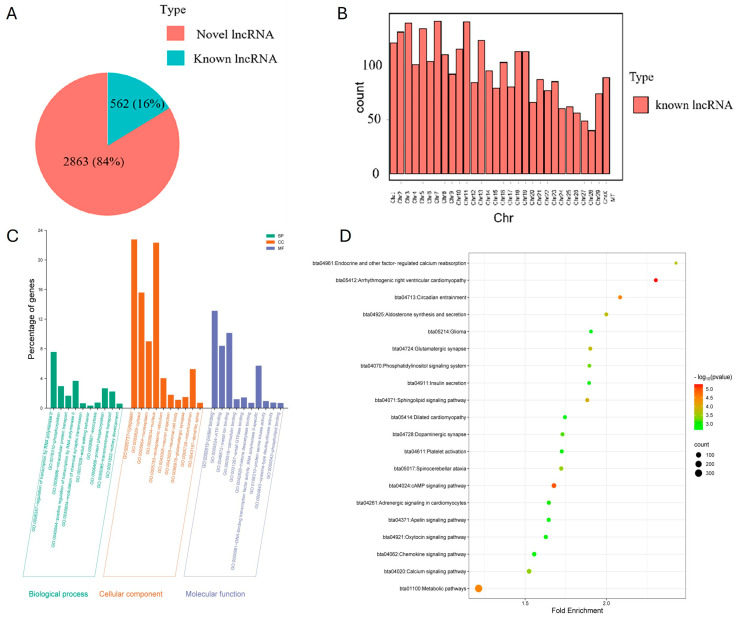
LncRNA-seq of adipocyte-derived exosomes: (**A**) Number of lncRNAs classified as “Novel” or “Known” (indicating whether lncRNAs are present in the NCBI database). (**B**) Distribution of lncRNAs across chromosomes. (**C**) Functional enrichment analysis of target genes. (**D**) Pathway enrichment analysis of target genes.

**Figure 6:**
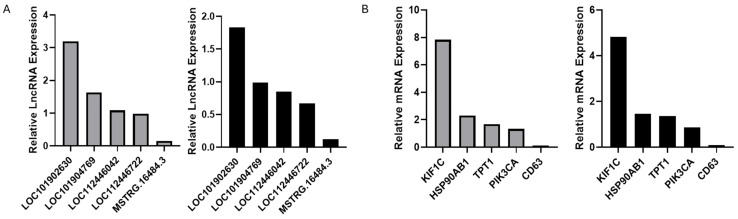
(**A**) The lncRNA sequencing results (predicted lncRNA expression levels) and RT-qPCR validation results (actual lncRNA expression levels). (**B**) The mRNA sequencing results (predicted mRNA expression levels) and RT-qPCR validation results (actual mRNA expression levels).

After quality control and filtering of the sequencing data, we obtained a total of 31,425,139 clean readings (Table S2). The Q30 value was higher than 91.49% and the successful comparison of total mapped reads was 97.11%. The identification of lncRNAs was performed by four software packages, and a total of 3425 lncRNAs were identified, of which 2863 were known lncRNAs, and 562 were novel lncRNAs (Figure 5A and Table S3). In addition, the annotated and screened lncRNAs were distributed throughout the bovine genome (Figure 5B). Based on the co-localization and co-expression of lncRNAs and protein-coding genes, we predicted 3835 genes as the target genes for the identified lncRNAs (Tables S4 and S5). We performed functional enrichment analysis of lncRNA target genes, and the results showed that a total of 469 GO entries and 98 KEGG pathways were enriched (Figure 5C,D and Table S6). The top 10 biological processes, such as transcriptional regulation, transmembrane transport, and phosphorylation, were significantly enriched in the BP category. Some terms, such as cytoplasm, nucleoplasm, and organelle parts, appeared in the top 10 CC categories. And the top 10 terms in the MF category were functionally enriched in relation to protein binding, DNA-binding transcription factor activity, RNA polymerase II-specific, and phospholipid binding. The first 20 pathways analyzed by KEGG showed that the target genes of lncRNAs were enriched in the cAMP signaling pathway, metabolic pathways, aldosterone synthesis and secretion, calcium signaling pathway, and other pathways. In order to verify the accuracy of the lncRNA sequencing results, five lncRNAs with different expressions, as well as five mRNAs, were randomly selected for RT-qPCR verification in this study. The results showed that they had the same expression trend, indicating that the sequencing results were reliable (Figure 6A,B).

### 3.6. Bioinformatics Analysis and Functional Validation of lncDGAT2

**Figure 7:**
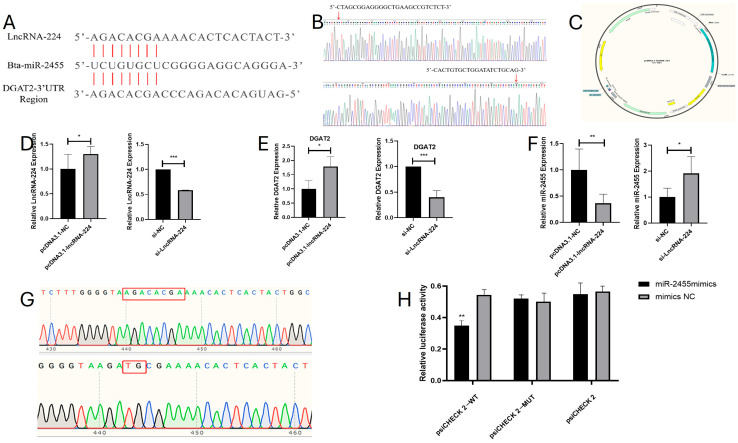
Bioinformatics analysis and functional validation: (**A**) Target binding site prediction for LncDGAT2, Bta-miRNA-2455, and DGAT2-3′UTR region. (**B**) Sequencing results of the lncDGAT2 vector. (**C**) Diagram illustrating the construction of the lncDGAT2 vector (the non-English term in center represents to “carbon-based pairs”). (**D**) Transfection efficiency of the lncDGAT2 vector. (**E**) Expression levels of DGAT2 under lncDGAT2 overexpression conditions and lncDGAT2 inhibition conditions. (**F**) Expression levels of bta-miR-2455 under lncDGAT2 overexpression conditions and lncDGAT2 inhibition conditions. (**G**) Wild-type plasmid (red box indicates predicted target site) and mutant-type plasmid (red box indicates mutation site). (**H**) Dual-luciferase assay for the targeting relationship between lncDGAT2 and bta-miR-2455; mean ± SEM, *n* = 3, “*”, “**” and “***” represent *p* < 0.05, *p* < 0.01 and *p* < 0.001, respectively.

In a previous study, we found that Bta-miR-2455 was associated with intramuscular fat deposition in cattle [39]. In this experiment, we found that DGAT2 is the predicted target gene of lnc224 (LOC100848315, lncDGAT2) based on the cis mode of action. Bioinformatics predictive analyses show that lncDGAT2 may have a targeting relationship with Bta-miR-2455, and DGAT2 is also the predicted target gene of Bta-miR-2455 (Figure 7A). LncDGAT2 was located on bovine chromosome 16 (55234405-55238240) with a total length of 3836 bp. Inter-species homology comparison of lncDGAT2 using the NCBI database showed that it was highly conserved only in the genomes of Bos Mutus, Ovis canadensis, and Cervus canadensis, with sequence homologies of 100%, 100%, and 98%, respectively. However, they showed low conservation in other species, which is in line with the biological characteristic of low conservation of lncRNAs among species.

Sequencing of the overexpression vector confirmed the successful construction of the lncDGAT2 carrier, with the synthesized lncDGAT2 transcript sequence fully integrated into the vector without any peaks or anomalies (Figure 7B,C). The overexpression vector for lncDGAT2 (pcDNA3.1-lncDGAT2), lncRNA-244 siRNA (si-lncDGAT2), and empty vector controls (pcDNA3.1-NC and si-NC) were separately transfected into MSCs, and RT-qPCR was performed to assess gene expression. The results showed that, compared to the pcDNA3.1-NC control group, the expression of lncDGAT2 was significantly upregulated in the pcDNA3.1-lncDGAT2 group (*p* < 0.05). Additionally, compared to the si-NC control group, the si-lncDGAT2 group exhibited a significant decrease in lncDGAT2 expression (*p* < 0.001), indicating successful transfection of these vectors into MSCs (Figure 7D). After transfecting pcDNA3.1-lncDGAT2, si-lncDGAT2, pcDNA3.1-NC, and si-NC into MSCs, total RNA was extracted 48 h later to measure the expression levels of the target genes DGAT2 and bta-miR-2455 (Figure 7E,F). Overexpression of lncDGAT2 led to a significant increase in DGAT2 expression (*p* < 0.05) and a highly significant decrease in bta-miR-2455 expression (*p* < 0.01). Conversely, inhibition of lncDGAT2 expression resulted in a highly significant decrease in DGAT2 expression (*p* < 0.01) and a significant increase in bta-miR-2455 expression (*p* < 0.05).

To determine whether lncDGAT2 directly targets bta-miR-2455, a fragment containing the seed sequence-binding site of bta-miR-2455 within lncDGAT2 was amplified. The wild-type recombinant plasmid psiCHECK 2-WT and mutant-type recombinant plasmid psiCHECK 2-MUT (Figure 7G) were constructed. HepG2 cells were co-transfected with bta-miR-2455 mimics or negative controls with differently treated dual-luciferase reporter plasmids. After 24 h, the luciferase activity was measured in the different transfection groups (Figure 7H). Transfection with bta-miR-2455 mimics significantly reduced the fluorescence activity of the wild-type recombinant plasmid (*p* < 0.05) but had no inhibitory effect on the mutant and empty vector plasmids. Negative control transfection had no significant effect on any of the plasmids (*p* > 0.05). These results suggest that lncDGAT2 competitively binds to bta-miR-2455, thereby affecting the expression of its target gene DGAT2.

## 4. Discussion

Intramuscular fat content, especially in the longissimus dorsi muscle, is a key determinant of beef quality and value [40]. Intramuscular fat deposition is a continuous and complex process that is influenced by genetic, nutritional, and physiological factors [38]. MSCs have been found to be induced to differentiate into a variety of cellular forms and to be a source of intramuscular fat formation [41]. Nevertheless, the mechanisms through which adipocytes promote the adipogenic transdifferentiation of MSCs remain less extensively investigated. To address this knowledge gap, we established a co-culture system of bovine adipocytes and MSCs. Using this model, we aimed to identify adipose-derived functional molecules implicated in adipogenic transdifferentiation of MSCs and to elucidate their potential molecular mechanisms and key regulatory pathways. Furthermore, leveraging lncRNA sequencing, we explored the involvement of an lncRNA-miRNA-mRNA regulatory network in adipogenesis.

In this study, we successfully established a co-culture system for bovine adipocytes and MSCs. The co-culture system can approximate the physiological environment of cells in vivo and the paracrine interactions between adipose tissue and muscle tissue under physiological conditions [42]. It serves as an effective tool for investigating the proliferation, differentiation, and function of two or more types of cells that interact in the physiological environment [43]. While interactions between muscle cells and adipocytes remain underexplored, co-culture systems are invaluable for such research. For example, Xu et al. successfully constructed a co-culture system of Tan sheep MSCs and preadipocytes, and found that Tan sheep MSCs could inhibit the lipid production of preadipocytes [44]. In contrast, our findings revealed that co-culture with bovine adipocytes upregulated the expression of key lipogenic genes and proteins and enhanced lipid deposition in bovine MSCs. Notably, MSCs can enter into lipogenic differentiation and transdifferentiate into adipocytes [45,46,47]. It was found that co-culture of chicken intramuscular preadipocytes promoted lipid deposition in MSCs [17], and bovine preadipocytes increased the expression of lipogenic genes in bovine MSCs [16], which was consistent with the results of this study. Collectively, these findings underscore the role of adipocytes in influencing the adipogenic transdifferentiation of MSCs and provide a rationale for subsequent investigation into the role of adipose-derived exosomes in this process.

Exosomes are carriers of intercellular substance exchange and signal transduction and contain biomolecules such as proteins, lipids, and genetic material [48]. However, the role of adipocyte-derived exosomes on MSCs is largely unknown. But it has been found that adipocytes can suppress the expression levels of muscle differentiation-related genes in myoblasts via exosomes [49]. In this study, we characterized extracellular vesicles derived from bovine adipocytes and found that they conformed to the typical features of exosomes in terms of morphology and expression of marker proteins [50,51]. In addition, the results of this study showed that MSCs co-cultured with adipocyte-derived exosomes increased the protein expression levels of PPARγ and CEBPA, the key genes for lipid formation. PPARγ belongs to the nuclear receptor family and can regulate the mRNA expression of multiple signaling pathway genes at the transcriptional level [52]. Moreover, the PPARγ signaling pathway is also a necessary transcription factor to regulate fat metabolism, which is at the core hub of lipid deposition signal transmission [53,54], and promotes lipid deposition by regulating the transcription of genes related to fat metabolism [55,56]. CEBPA is a key transcription factor in preadipocyte differentiation [57,58]. When the expression of CEBPA is inhibited, the transcription of glycogen synthase mRNA is delayed, which ultimately leads to the inability of adipocytes to accumulate lipids [59]. CEBPA and PPAR cross-regulate each other through positive feedback loops and trans-activate downstream target genes [60,61]. Together with CEBPβ, they form a cascade reaction axis that drives the terminal differentiation programme of adipocytes and influences adipose tissue development [62]. Moreover, a study has found that exosomes derived from adipocytes can enter adjacent adipocytes to promote lipid droplet or adipogenesis [63]. Indrakusuma et al. reported that factors secreted by adipocytes may induce insulin resistance in skeletal muscle cells [64]. Therefore, adipocytes might exert their effects on MSCs by secreting adipose-derived exosomes, leading to increased expression of adipogenic genes within MSCs, and inducing adipogenic transdifferentiation of MSCs by intervening in the PPAR signaling pathway and the CEBP cascade reaction axis.

However, it is worth noting that the protein expressions of PPARγ and CEBPA in MSCs co-cultured with adipocyte-derived exosomes were lower than those co-cultured with adipocytes. Intercellular interactions are dynamic and complex, involving the participation of many different factors [65]. Therefore, the phenomenon of lipogenic transdifferentiation of MSCs is a result of the combined action of many factors secreted by adipocytes, and exosomes are only one of the factors leading to the upregulation of the expression of lipogenic genes, which may also contribute to the above phenomenon.

In order to further investigate the potential mechanism of adipocyte-derived exosome mediation, we applied RNA-seq analysis to screen potential lncRNAs that influence the lipogenic transdifferentiation of MSCs. Several studies have reported that lncRNAs are associated with intramuscular fat deposition and metabolism [14,66,67]. Relatively speaking, the role of exosome lncRNAs in the process related to intramuscular fat deposition has received little attention. In this study, we found that abundant lncRNAs in adipocyte-derived exosomes. Moreover, the role of exosome lncRNAs in the intercellular communication mechanism has been revealed [68]. We performed GO and KEGG analyses of cis-acting target genes of exosomal lncRNAs and found that these genes exert functions such as transcriptional regulation, transmembrane transport, protein binding, DNA-binding transcription factor activity, and participate in the cAMP signaling pathway, metabolic pathways, phosphatidylinositol signaling system, and insulin secretion. Among them, the cAMP signaling pathway is one of the most well-defined mechanisms controlling adipocyte differentiation and plays an important role in lipogenesis and metabolism [69]. The phosphatidylinositol signaling system is not only an important pathway for regulating cell differentiation, but also regulates cellular lipid distribution and metabolism through its close relationship with lipid transporter proteins [70]. The insulin secretion is inextricably linked to lipid production and metabolism [71]. It was found that cis-regulated lncRNAs have an enhancer-like activity that promotes the expression of neighboring genes [72]. Therefore, the function of lncRNAs can be inferred from knowledge of their target genes. This suggests that adipocyte-derived exosome lncRNAs may play a role in transcriptional regulation and participate in cell differentiation, lipid production, and metabolism-related pathways.

LncRNAs are key regulators in the control of gene expression [73] and can regulate gene expression through cis-acting [74]. In addition, lncRNAs can act as ceRNA sponges to adsorb miRNAs on their homologous targets, thus alleviating the inhibition of target genes by miRNAs [75]. This study found that DGAT2 is cis-regulated by lncDGAT2, and lncDGAT2 can affect the expression of the target gene DGAT2 through competitive binding to bta-miR-2455. DGAT2 is involved in TG synthesis and affects the formation of lipid droplets [76,77,78]. Another study has shown that overexpression of DGAT2 can promote the differentiation of bovine preadipocytes and lipid droplet production and triacylglycerol accumulation in bovine preadipocytes [79]. In addition, overexpression of DGAT2 promotes lipid droplet formation and triacylglycerol accumulation in bovine MSCs, and DGAT2 plays a role in regulating the process of adipogenic transdifferentiation of bovine MSCs [80]. Notably, this study found that DGAT2 gene expression was significantly increased when lncDGAT2 was overexpressed in MSCs. Therefore, bovine adipocyte-derived exosomes can increase the expression of the target gene DGAT2 by increasing the expression of lncDGAT2 in bovine MSCs, thus promoting lipid synthesis in MSCs and regulating adipogenic transdifferentiation of MSCs.

The mechanisms governing muscle–fat crosstalk have garnered significant interest in recent years. Extensive research has identified numerous mediators—including cytokines, metabolites, and exosomes—that are involved in the interaction between muscle and fat [81]. For example, the adipokines leptin and adiponectin influence skeletal muscle metabolism: leptin promotes fatty acid oxidation and glucose uptake [82]. Adiponectin can play a role through autocrine/paracrine pathways. Overexpression of adiponectin can significantly upregulate genes related to mitochondrial fatty acid transport, increase lipid oxidation in skeletal muscle, and lead to the transformation of muscle [83]. Exosomes are a new type of carrier for intercellular communication. The specific material components contained in exosomes have the characteristics of functionality and targeting, indicating that they can play a role in regulating intercellular communication [84]. In this study, we provided experimental evidence that adipose-derived exosomes induce adipogenic transdifferentiation in MSCs by modulating the PPAR signaling pathway and the C/EBP cascade, thereby establishing a role for exosome-mediated communication from bovine adipocytes to MSCs. In the study of exosomes involved in muscle–adipose tissue interaction, researchers often focus on exosomal miRNAs. For example, Yu et al. found that adipocyte-derived exosomal miR-27a induces insulin resistance in skeletal muscle through repression of PPARγ [22]; Qin et al. found that skeletal muscle-derived exosomal miR-146a-5p inhibits adipogenesis by mediating the muscle–fat axis and targeting GDF5-PPARγ signaling [85]. Unlike these studies, we constructed an lncDGAT2-bta-miR-2455-DGAT2 regulatory network involved in adipogenesis by LncRNA-seq of exosomes and verified the role of exosome LncRNA in muscle–fat interaction. It is important to note that Qin et al. employed a combination of in vitro (Transwell) and in vivo (knockout model) approaches to rigorously establish the role of exosomal miR-146a-5p [85]. In contrast, our present findings are derived from in vitro co-culture models. Therefore, although our work elucidates a novel exosomal lncRNA-mediated mechanism for adipocyte–MSC crosstalk, future validation using animal models is essential to confirm its physiological relevance in vivo.

## 5. Conclusions

In summary, this study demonstrates that co-culture with bovine adipocytes induces adipogenic transdifferentiation in bovine muscle satellite cells (MSCs). We further established that adipose-derived exosomes are key mediators of this process, which function by modulating the PPARγ signaling pathway and the C/EBP transcriptional cascade. Moreover, we identified and functionally validated a novel ceRNA network—lncDGAT2/bta-miR-2455/DGAT2—that promotes adipogenesis in MSCs, thereby elucidating a specific mechanism by which exosomal lncRNAs regulate muscle–fat crosstalk. Collectively, our findings provide novel insights into the molecular interplay between adipose and muscle tissues and establish a valuable genetic framework for understanding the regulatory mechanisms of intramuscular fat deposition in beef cattle, with potential implications for improving meat quality.

## Figures and Tables

**Table 1:** RT-qPCR reaction system.

Reagent	Dosage	Concentration
SuperReal PreMix Plus (2×)	5 μL	1×
PCR Forward Primer (10 μM)	0.3 μL	0.3 μM
PCR Reverse Primer (10 μM)	0.3 μL	0.3 μM
cDNA	1 μL	
ROX Reference Dye (50×)	0.2 μL	1×
ddH_2_O	3.2 μL	
total	10 μL	

## Data Availability

The original contributions presented in this study are included in the article. Further inquiries can be directed to the corresponding author.

## Supplementary Materials

The following supporting information can be downloaded at: https://www.mdpi.com/article/10.3390/ani15233459/s1, Table S1: Primer information; Table S2: Clean Reads of LncRNA-seq; Table S3: LncRNAs information; Table S4: mRNAs information; Table S5: Target gene information for LncRNAs; Table S6: Result of KEGG and GO.

## Abbreviations

CD81: cluster of differentiation 81 receptor; CEBPA: CCAAT/enhancer binding protein alpha; DGAT2: Acyl CoA: diacylglycerol acyltransferase 2; DMEM: Dulbecco’s modified Eagle medium; DMSO: dimethyl sulfoxide; IMF: intramuscular fat; IBMX: 3-isobutyl-1-methyl-7H-xanthine; lncRNAs: long non-coding RNAs; IPGTT: intraperitoneal glucose tolerance test; IPITT: intraperitoneal insulin tolerance test; miRNAs: microRNAs; mRNA: messenger RNA; MSCs: muscle satellite cells; MyoD: Myogenic Differentiation Antigen; MyoG: Myogenin; Myf5: Myogenic factor 5; MSTN: Myostatin; NTA: nanoparticle tracking analysis; PPARγ: peroxisome proliferator-activated receptor γ; PBS: phosphate-buffered saline; RNA-Seq: RNA sequencing; TG: triglyceride; UTR: untranslated region.
